# Biochemical analysis of hatchet self-cleaving ribozymes

**DOI:** 10.1261/rna.052522.115

**Published:** 2015-11

**Authors:** Sanshu Li, Christina E. Lünse, Kimberly A. Harris, Ronald R. Breaker

**Affiliations:** 1 Howard Hughes Medical Institute, Yale University, New Haven, Connecticut 06520-8103, USA; 2 Department of Molecular, Cellular and Developmental Biology, Yale University, New Haven, Connecticut 06520-8103, USA; 3 Department of Molecular Biophysics and Biochemistry, Yale University, New Haven, Connecticut 06520-8103, USA

**Keywords:** comparative sequence analysis, phosphoester transfer, phosphorothioate, RNA processing, RNA cleavage

## Abstract

Hatchet RNAs are members of a novel self-cleaving ribozyme class that was recently discovered by using a bioinformatics search strategy. The consensus sequence and secondary structure of this class includes 13 highly conserved and numerous other modestly conserved nucleotides interspersed among bulges linking four base-paired substructures. A representative hatchet ribozyme from a metagenomic source requires divalent ions such as Mg^2+^ to promote RNA strand scission with a maximum rate constant of ∼4 min^−1^. As with all other small self-cleaving ribozymes discovered to date, hatchet ribozymes employ a general mechanism for catalysis involving the nucleophilic attack of a ribose 2′-oxygen atom on an adjacent phosphorus center. Kinetic characteristics of the reaction demonstrate that members of this ribozyme class have an essential requirement for divalent metal ions and that they might have a complex active site that employs multiple catalytic strategies to accelerate RNA cleavage by internal phosphoester transfer.

## INTRODUCTION

Self-cleaving ribozymes that function by internal phosphoester transfer are the most diverse of the various classes of RNA enzymes found in nature (Ferré-D'Amaré and Scott 2010). They comprise nine of the 14 different ribozyme classes that have been discovered to date. Among these are three self-cleaving ribozyme classes discovered only recently (Weinberg et al. 2015) by exploiting the fact that some of the common self-cleaving ribozymes such as hammerhead (Perreault et al. 2011; Hammann et al. 2012), HDV (Webb and Lupták 2011), and twister (Roth et al. 2014) frequently reside in the noncoding regions near particular types of genes. Specifically, a bioinformatics pipeline that employed comparative sequence and structural analysis algorithms was used to search for novel structured RNAs in a collection of noncoding regions near genes commonly associated with known self-cleaving ribozymes.

One of these newly found ribozymes, called hatchet, was shown to undergo site-specific self-cleavage in the presence of Mg^2+^ ions (Weinberg et al. 2015). In this Report, we present additional evidence for ribozyme function and provide a more detailed description of the biochemical properties of a hatchet RNA. Structural and kinetic characteristics of two bimolecular hatchet ribozyme constructs have been examined, and our findings confirm that this collection of RNAs merit a classification that is distinct from all previously known natural and engineered ribozyme classes. Furthermore, some of the kinetic properties of hatchet ribozymes are helpful when speculating on the catalytic strategies used by this collection of RNAs to promote RNA strand scission. The hatchet constructs tested in this study have modest rate constants compared to those exhibited by members of other self-cleaving ribozyme classes. However, our findings suggest that the active site formed by this ribozyme class might be complex, and therefore some representatives might be capable of promoting high-speed RNA cleavage.

## RESULTS AND DISCUSSION

### Updated consensus sequence and structural model for hatchet ribozymes

Previously, a consensus sequence and secondary structure model was created based on the alignment of 159 hatchet RNA representatives located entirely within metagenomic DNA sequences (Weinberg et al. 2015). After more exhaustive searches for similar RNAs, we identified a total of 210 representatives. However, no examples have been found in organisms whose genomes have been completely sequenced.

**FIGURE 1.**
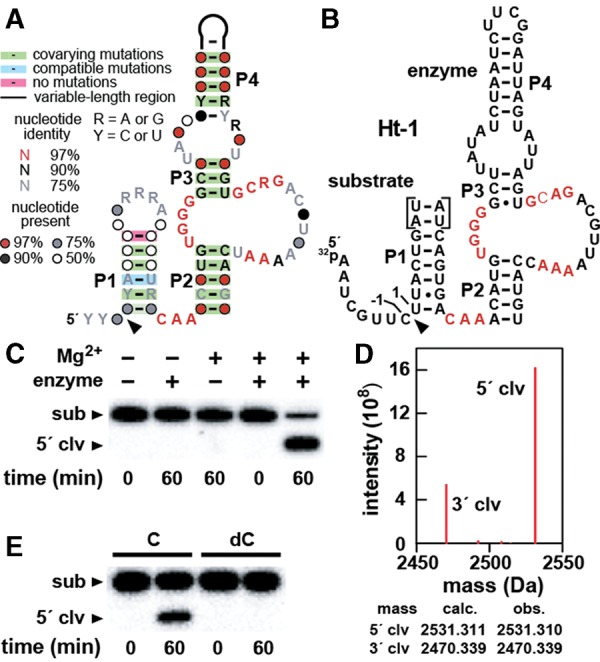
Sequence, structure, and function of hatchet ribozymes. (*A*) Updated consensus sequence and secondary structure model for hatchet ribozymes based on 210 different representatives. The arrowhead defines the site of ribozyme-mediated cleavage. Image was prepared using the R2R program (Weinberg and Breaker 2011). (*B*) Sequence and secondary structure of the bimolecular RNA construct Ht-1 based on metagenomic sequence SRS017191_Baylor_scaffold_14517/1281–1081 except that the loop region of P4 was replaced by UUCG. Nucleotides in red correspond to the most highly conserved positions in the consensus sequence. Nucleotides forming the 5′ and 3′ flanks of the cleavage site are numbered −1 and 1, respectively. Note that two non-native base pairs were added to P1 (brackets) to facilitate detection of the 3′ cleavage fragment by mass spectrometry. (*C*) Confirmation of ribozyme activity of Ht-1 carrying the two additional base pairs in P1. 5′ ^32^P-labeled substrate (∼5 nM) was incubated in the absence (−) or presence (+) of enzyme (∼100 nM) either in the absence or presence of 20 mM Mg^2+^ as indicated for each lane of the 20% PAGE gel. Additional details are described in Materials and Methods. The bands corresponding to the 16-nt substrate (sub) and the 8-nt 5′-cleavage product (5′-clv) are annotated accordingly. (*D*) Mass spectrometry analysis of a Ht-1 reaction depicting peaks corresponding closely with the calculated masses for 5′-clv and the corresponding 8-nt 3′-cleavage product annotated 3′-clv. The calculated (calc.) and observed (obs.) atomic masses for the cleavage products are presented. (*E*) Analysis of a Ht-1 construct with a 6-bp P1 and a 2′deoxyribonucleotide (dC) replacing the cytidine ribonucleotide (C) at position −1 of the substrate. Other annotations are as described in *C*.

A revised consensus model for hatchet ribozymes (Fig. 1A) carries all the key features of the initial model. The secondary structure includes four major base-paired substructures, called P1 through P4, and an additional P0 hairpin that is functionally dispensable (see below). Although P1 is depicted with six base pairs, hatchet RNAs almost always have only three (40%) or six (59%) base pairs forming this stem. This suggests that, in their natural settings, these precise lengths for P1 are important for the function of hatchet ribozymes.

The additional hatchet examples retain all but one of the nucleotides originally determined to be present in 97% or more of the representatives. These conserved nucleotides are positioned in the linker between P1 and P2, and in the asymmetric internal bulge that bridges P2 and P3. Conserved nucleotides in these positions likely participate in organizing the active site for RNA cleavage, which occurs at the base of P1.

### Hatchet RNAs are self-cleaving ribozymes

Contiguous hatchet RNA constructs undergo efficient self-cleavage during preparation by transcription in vitro (data not shown). To prevent uncontrolled ribozyme cleavage, we previously conducted self-cleavage activity assays by using bimolecular RNA constructs (Weinberg et al. 2015), which were created by splitting the RNA motif either in the loop of P1 or in the loop of P4. The RNA strand carrying the cleavage site was defined as the substrate, whereas the strand carrying the remainder of the key RNA sequence and structural elements was defined as the enzyme. For the current study, two versions of construct Ht-1 (Fig. 1B) were created by deleting the loop of P1 and forming constructs using two RNA strands wherein the substrate is composed of either 14 (short construct) or 16 (long construct) nucleotides (nt). Additionally, the P4 stem-loop of Ht-1 constructs was truncated by the replacement of 44 natural nucleotides with a UUCG tetraloop, which was expected to promote formation of the shortened P4 substructure.

The long version of the Ht-1 bimolecular complex cleaves the ^32^P-labeled substrate strand only when Mg^2+^ is added to the reaction mixture (Fig. 1C). To confirm the location of the cleavage site that was previously established by gel electrophoresis mobility (Weinberg et al. 2015), mass spectrometry was used to analyze the cleavage products (Fig. 1D). As previously suggested, the mass peaks confirm that the substrate is cleaved to yield a 5′ product with a terminal 2′,3′-cyclic phosphate group and a 3′ product with a 5′-hydroxyl group. Therefore, hatchet ribozymes likely use the same mechanism exploited by all other small self-cleaving ribozymes known, namely, internal phosphoester transfer (Emilsson et al. 2003; Ferré-D'Amaré and Scott 2010; Roth et al. 2014).

In this instance, the 2′ oxygen of the C at position −1 of Ht-1 attacks the adjacent phosphorus atom that results in the departure of the 5′ oxygen of U at position 1 (Fig. 1B). This general mechanism for hatchet action is also supported by the observation that the removal of the putative nucleophile causes a complete loss of activity. Specifically, when the C at position −1 of the substrate strand is replaced with a 2′-deoxyribose C in the short Ht-1 construct, the enzyme strand is rendered inactive (Fig. 1E).

About 90% of hatchet ribozymes have an additional hairpin structure, called P0, located on the 5′ side of the cleavage site, usually with only a few nucleotides separating P0 and P1 (Weinberg et al. 2015). This hairpin lacks evidence of sequence conservation, and therefore its role in promoting ribozyme function is not apparent. Hammerhead ribozymes found in nature are known to carry accessory domains near stems I and II that form tertiary contacts and that promote high-speed ribozyme function (Canny et al. 2004; Perreault et al. 2011). However, the results with Ht-1 constructs and others examined in this study (see below) suggest that P0 structures are not critical for ribozyme function. Therefore, all biochemical experiments presented herein are conducted with constructs that lack P0.

### The importance of conserved sequence and secondary structure features

**FIGURE 2.**
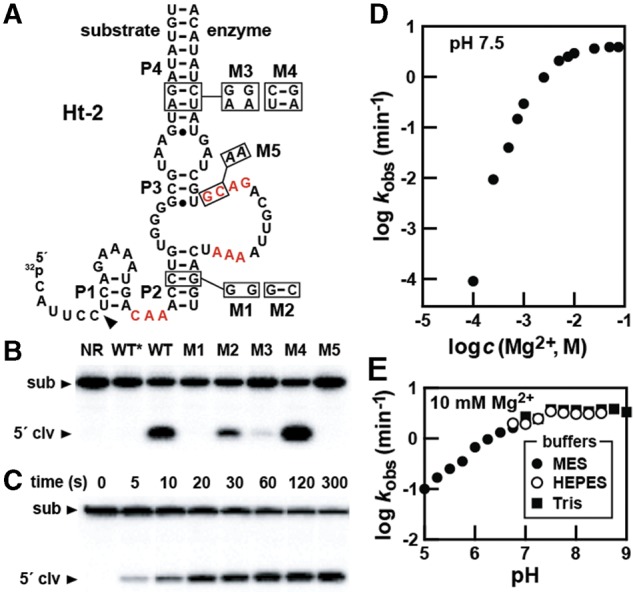
Structure and activity assays with hatchet construct Ht-2. (*A*) Sequence and secondary structure for the bimolecular construct Ht-2 derived from a metagenomic sequence. Mutations (M1, M3, and M5) and compensatory mutations (M2 and M4) that restore structure are boxed and occur at the locations indicated. Other annotations are as described in the legend to Figure 1B. (*B*) Ribozyme activities of WT or mutant Ht-2 constructs using a 5′ ^32^P-labeled substrate RNA. (NR) The reaction with WT RNA at time 0 as prepared by adding stop buffer before the addition of Mg^2+^. The asterisk denotes a reaction mixture without added enzyme strand. Other annotations and reaction conditions are as described in the legend to Figure 1C. (*C*) An example of a time course assay for ribozyme cleavage used to establish a *k*_obs_ value under a single reaction condition. (*D*) Plot representing the dependence of *k*_obs_ on Mg^2+^ concentration. (*E*) Plot representing the dependence of *k*_obs_ on pH. Three different buffers were used to span the pH range from 5 to 9.

To further evaluate the secondary structure model for hatchet ribozymes and to assess the importance of certain conserved nucleotides, we employed another metagenomic bimolecular hatchet ribozyme construct called Ht-2 (Fig. 2A). This construct was split into two parts at P4 because the P1 stem, which is formed with only three base pairs, was considered too weak to support bimolecular ribozyme formation by deleting the loop of P1. The ribozyme activity of the wild-type (WT) Ht-2 RNA construct proved to be robust, and therefore bimolecular versions of this RNA and various mutant constructs were used for most of the kinetic analyses in this study.

For example, Ht-2 ribozyme self-cleavage assays revealed that disruption of either P2 (M1) or P4 (M3) causes a loss of activity, whereas compensation mutations that restore base-pairing (M2 and M4) restore ribozyme activity (Fig. 2B). In addition, the mutation of two highly conserved nucleotides (M5) results in an inactive construct. Thus, the various Ht-2 constructs exhibit levels of activity that are consistent with the anticipated importance of the proposed secondary structure features and conserved nucleotides (Fig. 1A).

### The effects of pH and Mg^2+^ on hatchet ribozyme activity

The importance of Mg^2+^ concentration and pH on ribozyme activity was also assessed using the WT Ht-2 construct. These analyses were conducted by establishing a rate constant for substrate cleavage under each condition evaluated. For each reaction, ribozyme catalysis was initiated by the addition of MgCl_2_ and terminated at each time point by the addition of a stop solution containing urea and EDTA. The reaction products were separated by denaturing (8 M urea) 20% polyacrylamide gel electrophoresis (PAGE) (e.g., see Fig. 2C).

A plot of the *k*_obs_ values measured at pH 7.5 with increasing concentrations of Mg^2+^ (Fig. 2D) reveals a sharp increase in ribozyme function that plateaus as the concentration approaches 10 mM. The steep slope observed at lower Mg^2+^ concentrations suggests that more than one metal ion is necessary for each RNA to achieve maximal ribozyme activity. Moreover, this plot suggests that the construct requires higher than physiological concentrations of Mg^2+^ to become saturated with this divalent metal ion cofactor. Therefore, it is possible that native unimolecular constructs, perhaps also carrying P0, might achieve metal ion saturation at concentrations of Mg^2+^ that are more physiologically relevant.

We subsequently evaluated the effect of pH on ribozyme rate constant in reactions containing 10 mM Mg^2+^, which is presumably near saturation for the Ht-2 ribozyme construct used. pH-dependent ribozyme activity increases linearly with a slope of 1 until reaching a *k*_obs_ plateau of ∼4 min^−1^ near a pH value of 7.5. Both the pH dependency and the maximum rate constant exhibited by Ht-2 have interesting implications for the possible catalytic strategies used by this ribozyme class, as further discussed below.

### The effects of various mono- and divalent metal ions on hatchet ribozyme activity

As seen with the Ht-2 construct, steep Mg^2+^-dependent *k*_obs_ profiles also have been observed for the recently discovered twister (Roth et al. 2014) and twister sister (Weinberg et al. 2015) ribozymes. One atomic-resolution structure model of twister reveals the coordination of four Mg^2+^ ions in the folding of the twister RNA (Liu et al. 2014). None of these metal ions reside at the active site of twister, but rather participate with RNA to form the global fold of the ribozyme. Therefore, the large dependency of hatchet ribozymes on Mg^2+^ also could be purely structural, rather than an indication of direct participation of metal ions in catalysis. However, another structural model places a Mg^2+^ ion in coordination with the cleavage site phosphate moiety (Ren et al. 2014), but this metal ion is absent in an additional structural model of the twister ribozyme active site (Eiler et al. 2014). Given these uncertainties, the precise roles played by divalent cations cannot yet be established for twister ribozymes despite considerable biochemical and structural data.

**FIGURE 3.**
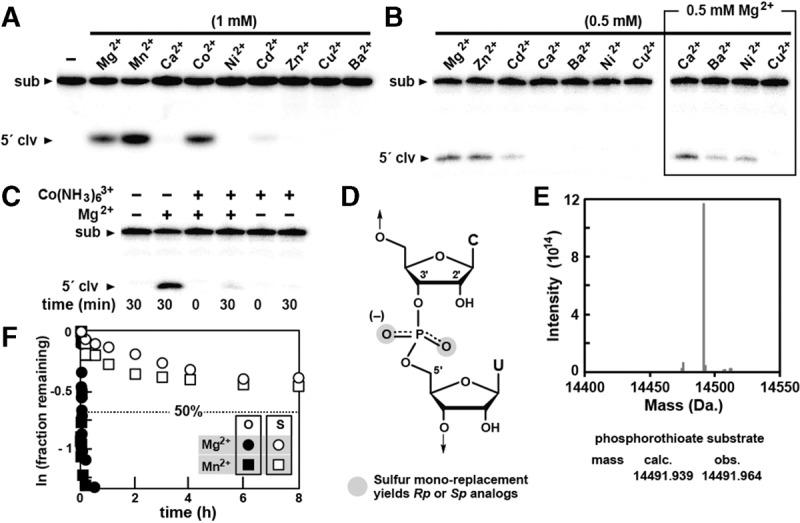
The effect of diverse metal ions on hatchet ribozyme activity. (*A*) Single time-point assays evaluating the ability of various divalent metal ions to support Ht-2 ribozyme activity. Reactions were incubated with 1 mM of the divalent metal ion as indicated for 30 min. Other experimental details and annotations are as described in the legend to Figure 1C. (*B*) Ht-2 ribozyme activity with certain divalent metal ions at 0.5 mM in the absence or presence of Mg^2+^. Other details are as described in Figure 1A. (*C*) The effect of cobalt hexammine on the function of Ht-2. Reactions were incubated in the absence (−) or presence (+) of 5 mM [Co(NH_3_)_6_]^3+^ and/or 5 mM Mg^2+^ as indicated. Other experimental details and annotations are as described in the legend to Figure 1C. (*D*) Sites of sulfur substitution (shaded) to generate the *Rp* or *Sp* isoforms of the ribozyme substrate. (*E*) Mass spectrometry data including the calculated (calc.) mass of the phosphorothioate substrate and the observed (obs.) mass of the sample. (*F*) Plot of the natural logarithm of the fraction of substrate RNA remaining uncleaved versus incubation time. O and S designate unmodified and phosphorothioate substrate RNAs, respectively. Of note, 10 mM MgCl_2_ or 10 mM MnCl_2_ were present in the reaction mixtures. Other conditions were as described in the legend to Figure 1C.

To further investigate the importance of Mg^2+^ and other ions for hatchet ribozyme catalysis, we conducted single time-point assays with various mono- and divalent ions. The Ht-2 construct remained completely inactive (data not shown) when incubated in the absence of Mg^2+^ in reactions containing various monovalent ions at 1 M (Na^+^, K^+^, Rb^+^, Li^+^, Cs^+^), 2.5 M (Na^+^, K^+^), or 3 M (Li^+^). In contrast, several other divalent metal ions such as Mn^2+^, Co^2+^, Zn^2+^, and Cd^2+^ support ribozyme function with varying levels of efficiency (Fig. 3A). Furthermore, two metal ions (Zn^2+^, Cd^2+^) function only at low concentrations, and three metal ions (Ba^2+^, Ni^2+^, and Cu^2+^) inhibit activity at 0.5 mM, even when Mg^2+^ is present (Fig. 3B). These results indicate that hatchet ribozymes are relatively restrictive in their use of cations to promote catalysis, perhaps indicating that one or more specialized binding sites that accommodate a limited number of divalent cations are present in the RNA structure or perhaps even at the active site. Inhibition by certain divalent metal ions could be due to the displacement of critical Mg^2+^ ions or by general disruption of RNA folding.

The nature of divalent metal ion binding sites in other self-cleaving ribozymes has been further probed by using cobalt hexammine, which is an inert metal complex with a size and hydrogen bonding potential similar to a hexahydrated magnesium ion (Cowan 1993; Hampel and Cowan 1997). If a ribozyme is activated by modest amounts of cobalt hexammine in the absence of otherwise essential divalent metal ions, this is usually interpreted as evidence against catalytic or structural metal ions with inner-sphere coordination to the RNA. For example, *glmS* (Roth et al. 2006) and twister (Roth et al. 2014) ribozymes are activated by cobalt hexammine, suggesting that they do not form essential inner-sphere metal ion contacts. This has been proven to be true for *glmS* ribozymes (Klein and Ferré-D'Amaré 2006; Cochrane et al. 2007). However, given the differences in the structural models for twister ribozymes (Eiler et al. 2014; Liu et al. 2014; Ren et al. 2014), it is not yet clear whether this ribozyme class requires inner-sphere coordination between Mg^2+^ and the RNA to exhibit robust self-cleavage activity. Also, the results of such assays with other self-cleaving ribozymes have sometimes produced findings that are difficult to interpret (e.g., see Curtis and Bartel 2001).

We found that cobalt hexammine at a concentration of 5 mM does not trigger Ht-2 ribozyme activity in the absence of Mg^2+^ (Fig. 3C), suggesting that hatchet ribozymes rely on one or more Mg^2+^ ions that form inner-sphere contacts with the RNA. However, cobalt hexammine almost completely inhibits ribozyme function even when a near-saturating amount of Mg^2+^ is present. Unfortunately, there are several possible explanations for these findings and therefore we cannot determine from these data whether hatchet ribozymes might use inner-sphere metal ion contacts or exclusively exploit fully hydrated Mg^2+^ ions to promote RNA cleavage.

### Reactivity of substrates carrying a phosphorothioate group at the ribozyme cleavage site

Mg^2+^ coordinates to oxygen ∼30,000-fold more strongly than to sulfur, whereas Mn^2+^ coordinates the two elements more equally (Jaffe and Cohn 1979; Pecoraro et al. 1984). As a result, a comparison of the reactivities of substrates carrying either a phosphate group or a phosphorothioate group (sulfur replacing one of the nonbridging oxygen atoms) at the cleavage site can be useful in determining whether there is a direct, inner-sphere coordination between a metal ion and a nonbridging atom at this location. If the ribozyme promotes RNA cleavage by coordinating with a nonbridging oxygen, then a hard metal ion such as Mg^2+^ would be expected to exhibit a substantial loss of ribozyme activity when sulfur is presented as a ligand. In contrast, a soft metal such as Mn^2+^ or Cd^2+^ should not suffer as great a loss in *k*_obs_ for the phosphorothioate substrate analog because of the higher affinity of these metal ions for sulfur (Dahm and Uhlenbeck 1991; Thaplyal et al. 2013).

A preliminary investigation into the effects of a phosphorothioate group at the site of hatchet ribozyme cleavage was conducted using the Ht-2 construct. A substrate analog population was prepared that is expected to have near equal amounts of the sulfur-containing *R*p and *S*p phosphorothioate isomers (Fig. 3D). The integrity of this phosphorothioate substrate analog was confirmed by mass spectrometry (Fig. 3E). This substrate preparation was incubated with enzyme strand and either Mg^2+^ or Mn^2+^, and compared to similar reactions with the normal oxygen-containing substrate. We observed that the normal substrate is rapidly cleaved by the enzyme strand in the presence of either Mg^2+^ or Mn^2+^ (1.5 min^−1^ and 4.6 min^−1^, respectively), and the substrate is processed to near completion. In stark contrast, the phosphorothioate substrate is processed much more slowly by both metals and the extent of the reaction is somewhat <50% (Fig. 3F).

These data suggest that perhaps one of the two isomers of the phosphorothioate substrate strongly resists cleavage by the ribozyme, while the other is cleaved far more slowly than the normal substrate. If true, this could be at least partly explained by the formation of a critical contact between a nonbridging phosphate oxygen and a moiety in the active site of the ribozyme. Curiously, the addition of Mn^2+^ does not functionally rescue this phosphorothioate substrate reactivity defect, which could be interpreted as a lack of inner-sphere metal ion coordination with a nonbridging phosphate oxygen in the unmodified ribozyme. However, additional experiments will be needed to determine the precise nature of the active site, and the possible involvement of metal ions in hatchet structure formation and catalysis.

### The catalytic strategies used by hatchet ribozymes

Self-cleaving ribozymes can use four different catalytic strategies to promote RNA cleavage (Emilsson et al. 2003). Specifically, enzymes that cleave RNA by internal phosphoester transfer can position the RNA linkage for in-line nucleophilic attack (called α), neutralize the negative charge on a nonbridging phosphate oxygen (β), deprotonate the 2′ oxygen nucleophile (γ), and neutralize the developing negative charge on the 5′-oxyanion leaving group (δ). These catalytic strategies presumably can be used either alone or in any combination to help stabilize the transition state of the reaction or otherwise promote cleavage. Since only these four catalytic strategies can contribute substantially to stabilization of the transition state stabilization, we do not consider transition state stabilization to be a separate distinct (or fifth) catalytic strategy. Since the kinetic characteristics and the maximum rate enhancements that can be derived by each of the four catalytic strategies have either been estimated or directly measured, there exists a simple framework for evaluating the function of different ribozyme classes (Emilsson et al. 2003).

Even with this framework in hand, establishing the precise strategies used by an enzyme can be challenging. This is true for hatchet ribozymes for the following reasons. Two kinetic features of the Ht-2 construct are similar to ribozymes that are predicted to use only α and γ catalytic strategies. The pH-reactivity profile (Fig. 2D) could be explained by a ribozyme with a critical functional group that has a p*K*_a_ near 7 and its extent of deprotonation limits the rate constant for RNA cleavage. The simplest explanation for this profile is that this key deprotonatable group is the 2′ oxygen nucleophile at the cleavage site, and that the ribozyme shifts its p*K*_a_ from its normal value of ∼13.7 (Li and Breaker 1999) to become near fully deprotonated at neutral pH. Even if this were true (there are many other possible explanations for such a pH profile), full deprotonation of the 2′-hydroxyl group would produce a maximum rate constant of ∼0.02 min^−1^. In other words, even if this ribozyme were fully exploiting the γ catalytic strategy, its *k*_obs_ value would still be about two orders of magnitude lower than the measured top speed of ∼4 min^−1^ for Ht-2.

Importantly, the internal phosphoester transfer reaction also requires proper in-line orientation of the attacking 2′-oxygen nucleophile, the phosphorus electrophilic, and the 5′-oxygen leaving group. RNAs with phosphodiester linkages positioned to some extent in an in-line orientation have been found to cleave with a rate enhancement of more than 10-fold above unstructured linkages (Usher and McHale 1976; Soukup and Breaker 1999). Furthermore, in-line geometry is unlikely to contribute much more than 100-fold to the rate enhancement of a catalyst (Soukup and Breaker 1999; Breaker et al. 2003; Min et al. 2007). Therefore, the rate enhancement that can be derived by a catalyst that fully employs α catalysis by perfectly pre-organizing the RNA linkage for in-line attack is very modest.

Intriguingly, ribozymes and deoxyribozymes created by directed evolution methods and predicted to only exploit α and γ catalytic strategies have maximum rate constants that approach 2 min^−1^ (Breaker et al. 2003). This also is near the theoretical limit for such enzymes assuming that the rate enhancements for α and γ are multiplicative (Emilsson et al. 2003). Both this “speed limit” and the pH profile of predicted αγ enzymes are very similar to that observed for Ht-2. Therefore, hatchet ribozymes could be maximally employing only α and γ catalytic strategies. Consistent with this hypothesis is the fact that we have examined bimolecular constructs based six different natural hatchet representatives, and none of these ribozymes exceed a rate constant of 4 min^−1^.

A major complication to this simple explanation is the fact that Ht-2 also exhibits some kinetic characteristics that are unlike those expected for αγ ribozymes. Ht-2 can process <50% of the phosphorothioate substrates in a reaction containing either Mg^2+^ or Mn^2+^ (Fig. 3F), suggesting that one of the two isomers of this substrate strongly interferes with catalysis. Ribozymes should not be strongly affected by a phosphorothioate group at the cleavage site unless they are forming a necessary contact with the nonbridging phosphate oxygen, and thereby inherently neutralizing the negative charge at the labile linkage. If true, this would mean that hatchet ribozymes indeed employ β catalysis, and that other more complex reasons are needed to explain the observed kinetic characteristics of this ribozyme class.

## CONCLUSIONS

Although the pace of discovery of novel ribozyme classes has been slow over the last 25 years, computational analyses of the rapidly increasing DNA sequence databases have recently yielded several new classes of self-cleaving ribozymes. Given the novel consensus sequence and structure of hatchet RNAs, and given the distinctive kinetic features of the representatives examined in this study, it is clear that they constitute a novel self-cleaving ribozyme class. As more genomes are sequenced, and as additional correlations are made between certain gene types and self-cleaving ribozymes, it is likely that the total number of distinct ribozyme classes will continue to increase.

Given the complexity of the initial kinetic data gathered in our current study, it is not yet possible to make definitive conclusions regarding the catalytic strategies used by hatchet ribozymes. A combination of more detailed kinetic studies and high-resolution structural analyses might be needed to reveal the true nature of an active site for this new ribozyme class. Regardless of the precise catalytic strategies used by hatchet ribozymes, it is striking that additional diversity of functional characteristics for self-cleaving ribozymes is being found, even though a total of nine natural self-cleaving ribozyme classes are already in hand. The structural and functional diversity of these RNAs, along with the diversity of biological roles they play, will be important to explore as more representative self-cleaving ribozymes are discovered.

## MATERIALS AND METHODS

### Bioinformatic discovery and analysis of hatchet ribozyme representatives

The 210 hatchet self-cleaving ribozyme representatives were discovered by using the computational algorithm Infernal (Weinberg et al. 2010; Nawrocki and Eddy 2013) to search the RefSeq (Pruitt et al. 2007) version 63 plus additional microbial data sets (Weinberg et al. 2015) for sequences that roughly correspond to the previously established consensus sequence and structure for this noncoding RNA class (Weinberg et al. 2015).

### Ribozyme self-cleavage assays

Cleavage assays were performed using conditions similar to those described previously (Roth et al. 2014). All substrate and enzyme RNAs were chemically synthesized (Sigma-Aldrich), with the exception of the enzyme strand for Ht-1, which was prepared by in vitro transcription (Roth et al. 2006) from a DNA template made by PCR using overlapping synthetic DNA primers (Forward primer: 5′-TAATACGACTCACTATAGGATCAGTGACAAACATGTGGGGCTTATATCTAATCTTCG; reverse primer: 5′-ACATGGTTTTAACGTCTGCACTAATACTAATCCGAAGATTAGATATAAGCCCCACAT). Substrate RNAs were enzymatically dephosphorylated with rAPid Alkaline Phosphatase (Roche Life Sciences) and 5′ ^32^P-labeled with T4 Polynucleotide Kinase (New England BioLabs) using conditions recommended by the manufacturers. Oligonucleotides were purified by denaturing 20% PAGE, isolated from the gel by crush-soaking in a solution containing 10 mM Tris–HCl (pH 7.5 at 23°C), 200 mM NaCl, and 1 mM EDTA, precipitated by the addition of 2.5 volumes of 100% cold ethanol, recovered by centrifugation, and resuspended in dH_2_O. Radiolabeled substrate and unlabeled enzyme RNAs were combined with a 2× stock solution of buffer in the absence of divalent metal ion. Five microliter aliquots of this mixture were delivered to separate tubes, heated for 1 min at 80°C, allowed to cool for 10 min at room temperature, and centrifuged briefly to eliminate condensation. Reactions were initiated by the addition of an equal volume of 2× metal ion stock solution. The final concentrations for the reagents were ∼5 nM substrate, 100 nM enzyme, 30 mM Tris–HCl buffer (pH 7.5 at 23°C), 100 mM KCl, and 20 mM MgCl_2_ (unless otherwise noted). The final volume of each reaction was 10 μL also unless otherwise noted. Reactions were incubated at 23°C for the times indicated for each experiment. Reactions were stopped by adding an equal volume of stop solution (90% formamide, 50 mM EDTA, 0.05% xylene cyanol and 0.05% bromophenol blue). The reaction products were separated by denaturing (8 M urea) 20% PAGE, imaged by using a STORM PhosphorImager (GE Healthcare) and quantified by using ImageQuant software.

### Observed rate constant (*k*_obs_) measurements

Measurements of *k*_obs_ values were carried out as described above using the bimolecular construct Ht-2. To establish the *k*_obs_ value for each reaction condition, a time course experiment from 5 sec to a few minutes or longer was conducted, and the products were separated and quantified as described above. The fraction of substrate RNA cleaved at each time point was corrected to account for the amount of substrate that remained intact even after exhaustive cleavage (typically <20% uncleaved). The *k*_obs_ for each reaction was derived from the time course data by using Prism software (GraphPad Software) with the equation

$$Y=Y_{0}+(P-Y_{0})(1-e^{{-k}_{\mathrm{obs}}t}),$$

where *Y* is cleavage fraction at time t, *k*_obs_ is observed rate constant, *Y*_0_ and *P* are the cleavage fraction at time 0 and the cleavage fraction after exhaustive incubation, respectively. This equation is based on those used previously to establish rate constants for self-cleaving ribozymes (Hendry et al. 1997; Roth et al. 2014)_._

### Mass spectrometry analysis of cleavage products

A 20 µL reaction containing 20 pmol each of Ht-1 substrate and enzyme was incubated in 30 mM HEPES (pH 7.5 at 23°C), 100 mM KCl and 20 mM Mg^2+^ for 1 h. The mixture was then subjected to monoisotopic (exact mass) spectrometry (Novatia).

### Analysis of cleavage characteristics of phosphorothioate substrates

The substrate of Ht-2 was chemically synthesized (Sigma-Aldrich) with a phosphorothioate moiety at the cleavage site. The isomeric mixture was purified, 5′ ^32^P-labeled, and purified again as described above. Both the unmodified substrate and the phosphorothioate substrate were incubated with the Ht-2 enzyme strand in reactions containing 10 mM MgCl_2_ or 10 mM MnCl_2_. Other conditions are the same as those used for standard cleavage assays.
